# Supplementary material for: Cross-subsidies are a viable option to fund formal pit latrine emptying services: Evidence from Kigali, Rwanda
Source: PLoS One. 2024 Aug 22;19(8):e0307471. doi: 10.1371/journal.pone.0307471 (PMC11341047; doi:10.1371/journal.pone.0307471)
Supplement: S1 File — (DOCX) [file pone.0307471.s002.docx]

# S1 Tables

**Table S1a. Pit Vidura annual direct costs, jobs, total revenue and average revenue organised by vehicle.** All financial values are in 2022 international dollars

| **Vehicle** | **Year** | **Total direct costs** | |  |  |  |  |  |  |  |  | **Total jobs per year** | **Total annual revenue** | **Average revenue per job** |
| --- | --- | --- | --- | --- | --- | --- | --- | --- | --- | --- | --- | --- | --- | --- |
|  |  | Fuel | Repair and maintenance | Vehicle depreciation | Vehicle rental | Emptiers' wages | Dumping fees | Drivers' salaries | Consumables | Equipment | Other |  |  |  |
| Large exhauster truck | 2018 | 0 | 0 | 0 | 0 | 0 | 0 | 0 | 0 | 0 | 0 | 0 | 0 | - |
| Large exhauster truck | 2019 | 1761 | 3939 | 11466 | 2774 | 988 | 295 | 0 | 85 | 171 | 263 | 36 | 8480 | 236 |
| Large exhauster truck | 2020 | 37329 | 26117 | 35512 | 14800 | 5548 | 4372 | 0 | 1077 | 1986 | 1447 | 255 | 115600 | 453 |
| Large exhauster truck | 2021 | 44825 | 33022 | 32918 | 8628 | 6067 | 7322 | 8373 | 2667 | 2165 | 3425 | 424 | 152602 | 360 |
| Large exhauster truck | 2022 | 81842 | 49087 | 16954 | 16758 | 11513 | 9117 | 9087 | 2438 | 2163 | 1001 | 442 | 211428 | 478 |
| Medium exhauster truck | 2018 | 1058 | 1346 | 1776 | 7382 | 2129 | 614 | 0 | 13 | 0 | 297 | 79 | 14079 | 178 |
| Medium exhauster truck | 2019 | 1025 | 13761 | 16506 | 13853 | 5389 | 1474 | 0 | 423 | 854 | 1314 | 226 | 40837 | 181 |
| Medium exhauster truck | 2020 | 17827 | 31854 | 17220 | 12501 | 7475 | 2235 | 0 | 910 | 1678 | 1222 | 345 | 66149 | 192 |
| Medium exhauster truck | 2021 | 20478 | 29813 | 15962 | 0 | 9309 | 2003 | 6784 | 2552 | 1704 | 2988 | 395 | 74122 | 188 |
| Medium exhauster truck | 2022 | 18080 | 13642 | 17324 | 67 | 3570 | 3071 | 3506 | 963 | 686 | 890 | 157 | 52939 | 337 |
| Small exhauster truck | 2018 | 0 | 0 | 0 | 0 | 0 | 0 | 0 | 0 | 0 | 0 | 0 | 0 | - |
| Small exhauster truck | 2019 | 0 | 0 | 0 | 0 | 0 | 0 | 0 | 0 | 0 | 0 | 0 | 0 | - |
| Small exhauster truck | 2020 | 0 | 0 | 0 | 0 | 0 | 0 | 0 | 0 | 0 | 0 | 0 | 0 | - |
| Small exhauster truck | 2021 | 915 | 1287 | 4911 | 0 | 64 | 0 | 660 | 1795 | 422 | 2803 | 75 | 10735 | 143 |
| Small exhauster truck | 2022 | 8491 | 6585 | 21143 | 796 | 3284 | 376 | 6690 | 1980 | 3049 | 1460 | 409 | 81923 | 200 |
| Semi-mechanical emptying | 2018 | 0 | 0 | 0 | 0 | 0 | 0 | 0 | 0 | 0 | 0 | 0 | 0 | - |
| Semi-mechanical emptying | 2019 | 0 | 0 | 0 | 0 | 0 | 0 | 0 | 0 | 0 | 0 | 0 | 0 | - |
| Semi-mechanical emptying | 2020 | 0 | 0 | 0 | 0 | 0 | 0 | 0 | 0 | 0 | 0 | 0 | 0 | - |
| Semi-mechanical emptying | 2021 | 429 | 604 | 2305 | 0 | 73 | 0 | 310 | 818 | 2539 | 1202 | 23 | 3208 | 139 |
| Semi-mechanical emptying | 2022 | 3042 | 2359 | 7575 | 285 | 2779 | 135 | 2397 | 710 | 3588 | 523 | 91 | 21509 | 236 |
| Flatbed truck | 2018 | 4587 | 5835 | 7695 | 31990 | 9226 | 2661 | 0 | 57 | 0 | 1288 | 321 | 54929 | 171 |
| Flatbed truck | 2019 | 15892 | 15305 | 0 | 17402 | 6940 | 1851 | 0 | 531 | 1073 | 1651 | 266 | 47843 | 180 |
| Flatbed truck | 2020 | 0 | 192 | 0 | 10633 | 12056 | 2035 | 0 | 774 | 1427 | 1039 | 295 | 54429 | 185 |
| Flatbed truck | 2021 | 0 | 0 | 0 | 17850 | 4476 | 543 | 0 | 1703 | 14351 | 1603 | 130 | 23592 | 181 |
| Flatbed truck | 2022 | 0 | 0 | 0 | 2538 | 970 | 0 | 0 | 269 | 676 | 0 | 19 | 2947 | 155 |

**Table S1b. Pit Vidura annual indirect costs.** All financial values are 2022 international dollars. Total costs includes direct costs.

| **Year** | **Staff salaries** | **Marketing and advertising** | **Staff expenses** | **Accounting, consulting and banking** | **Office** | **Tax** | **Communications** | **Other** | **Total** | **Proportion of total costs** |
| --- | --- | --- | --- | --- | --- | --- | --- | --- | --- | --- |
| 2018 | 17492 | 6305 | 23440 | 6747 | 6577 | 2469 | 1084 | 6445 | 70558 | 51% |
| 2019 | 117436 | 55504 | 35169 | 85709 | 14267 | 7737 | 8886 | 29545 | 354255 | 78% |
| 2020 | 115258 | 28080 | 12975 | 99524 | 13390 | 13201 | 12672 | 40054 | 335154 | 59% |
| 2021 | 89858 | 20880 | 14239 | 10982 | 18227 | 67958 | 14384 | 36271 | 272799 | 51% |
| 2022 | 81263 | 17133 | 9770 | 11068 | 27867 | 45293 | 11796 | 12635 | 216825 | 37% |

**Table S1c. Pit Vidura annual revenue organised by customer type, group and status.** All financial values are 2022 international dollars.*2016 and 2023 are partial years of two and six months respectively

| **Customer type** | **Customer group** | **Customer status** | **2016*** | **2017** | **2018** | **2019** | **2020** | **2021** | **2022** | **2023*** |
| --- | --- | --- | --- | --- | --- | --- | --- | --- | --- | --- |
| Household | Low-volume | First-time | 36023 | 33015 | 50528 | 42516 | 49453 | 28899 | 62336 | 72363 |
| Household | Low-volume | Repeat | 3409 | 3502 | 3815 | 4768 | 4154 | 8815 | 38978 | 54661 |
| Household | High-volume | First-time | 1136 | 13507 | 12414 | 35332 | 68532 | 74202 | 45063 | 54152 |
| Household | High-volume | Repeat | 0 | 1572 | 757 | 4484 | 4333 | 36438 | 35146 | 94263 |
| Corporate | - | First-time | 1136 | 1218 | 1494 | 10388 | 70416 | 35064 | 56415 | 29529 |
| Corporate | - | Repeat | 0 | 629 | 0 | 886 | 40651 | 87824 | 143941 | 252832 |
| Total | - | - | 41704 | 53442 | 69008 | 98373 | 237539 | 271242 | 381881 | 557800 |

**Table S1d. Pit Vidura average direct cost per job organised by emptying method and customer type.** All financial values are 2022 international dollars.

| **Emptying method** | **Customer** | **Year** | **Total jobs** | **Average direct cost per job** | **Total revenue** | **Proportion of annual revenue** |
| --- | --- | --- | --- | --- | --- | --- |
| Large exhauster truck | Corporate | 2019 | 21 | 684 | 5298 | 6% |
| Large exhauster truck | Corporate | 2020 | 157 | 622 | 95816 | 41% |
| Large exhauster truck | Corporate | 2021 | 237 | 387 | 102225 | 39% |
| Large exhauster truck | Corporate | 2022 | 267 | 495 | 135169 | 37% |
| Large exhauster truck | Household | 2019 | 15 | 492 | 3182 | 3% |
| Large exhauster truck | Household | 2020 | 98 | 311 | 19783 | 8% |
| Large exhauster truck | Household | 2021 | 187 | 308 | 50377 | 19% |
| Large exhauster truck | Household | 2022 | 175 | 388 | 76259 | 21% |
| Medium exhauster truck | Corporate | 2019 | 18 | 255 | 4204 | 4% |
| Medium exhauster truck | Corporate | 2020 | 55 | 277 | 13067 | 6% |
| Medium exhauster truck | Corporate | 2021 | 35 | 231 | 8116 | 3% |
| Medium exhauster truck | Corporate | 2022 | 46 | 432 | 25633 | 7% |
| Medium exhauster truck | Household | 2018 | 73 | 184 | 13171 | 20% |
| Medium exhauster truck | Household | 2019 | 208 | 240 | 36633 | 38% |
| Medium exhauster truck | Household | 2020 | 290 | 268 | 53081 | 23% |
| Medium exhauster truck | Household | 2021 | 360 | 232 | 66006 | 25% |
| Medium exhauster truck | Household | 2022 | 111 | 378 | 27306 | 7% |
| Small exhauster truck | Corporate | 2022 | 25 | 125 | 4117 | 1% |
| Small exhauster truck | Household | 2021 | 74 | 172 | 10374 | 4% |
| Small exhauster truck | Household | 2022 | 384 | 132 | 77805 | 21% |
| Semi-mechanical emptying | Household | 2021 | 23 | 360 | 3208 | 1% |
| Semi-mechanical emptying | Household | 2022 | 89 | 257 | 20629 | 6% |
| Semi-mechanical emptying | Household | 2018 | 318 | 197 | 54343 | 80% |
| Semi-mechanical emptying | Household | 2019 | 262 | 227 | 46993 | 49% |
| Semi-mechanical emptying | Household | 2020 | 292 | 95 | 53435 | 23% |
| Semi-mechanical emptying | Household | 2021 | 126 | 312 | 22256 | 8% |
| Semi-mechanical emptying | Household | 2022 | 19 | 234 | 2947 | 1% |

**Table S1e. Model assumptions (truck volumes, operating days per year and values from literature).** International dollars in 2022 (Int$).

| **Assumption** | **Value** | **Units** | **Notes** |
| --- | --- | --- | --- |
| Large exhauster truck volume | 20 | m^3^ | - |
| Medium exhauster truck volume | 10 | m^3^ | - |
| Small exhauster truck volume | 5 | m^3^ | - |
| Semi-mechanical emptying volume | 2 | m^3^ | - |
| Maximum jobs per day - mechanical emptying | 4 | jobs per day | - |
| Maximum jobs per day - semi-mechanical | 2 | jobs per day | - |
| Operating days per year | 250 | days per year | - |
| Proportion of households emptying or sealing pits | 87% | - | From literature^a^ |
| Tariff for households to use formal semi-mechanical emptying services | 24 | USD | From literature^a^ |
| Tariff for households to use formal semi-mechanical emptying services | 51 | Int$ | 63% reduction |
| Current tariff | 139 | Int$ | - |
| Mean household pit latrine emptying frequency | 8.7 | years | From literature^a^ |
| Mean low-income household size | 6.1 | people | From literature^a^ |
| Number households sharing a pit latrine | 4 | households | From literature^b^ |
| Kigali total population | 1,745,555 | people | From literature^c^ |
| Proportion of households using shared pit-latrines with constructed floor slabs | 46.90% | - | From literature^c^ |

^a^Burt Z, Sklar R, Murray A. Costs and Willingness to Pay for Pit Latrine Emptying Services in Kigali, Rwanda. Int J Environ Res Public Health. 2019;16(23):4738.

^b^Tsinda A, Abbott P, Pedley S, Charles K, Adogo J, Okurut K, et al. Challenges to Achieving Sustainable Sanitation in Informal Settlements of Kigali, Rwanda. International Journal of Environmental Research and Public Health. 2013;10(12):6939-54.

^c^NSIR. Fifth Population and Housing Census 2022. Kigali, Rwanda: National Insitute of Statistics of Rwanda; 2022.

**Table S1f. Emptying jobs completed during first 18 months of all three exhauster trucks being available – November 2021 to May 2023.**

| **Emptying method** | **Customer group** | **Jobs** |
| --- | --- | --- |
| Large exhauster truck | Corporate | 371 |
| Large exhauster truck | Household | 322 |
| Medium exhauster truck | Corporate | 79 |
| Medium exhauster truck | Household | 262 |
| Small exhauster truck | Corporate | 27 |
| Small exhauster truck | Household | 757 |
| Semi-mechanical emptying | Household | 0 |
| Flatbed truck | Household | 15 |
| Rental truck | Household | 5 |

**Table S1g. Pit Vidura annual gross and net profit – 2018 to 2022.** All financial values are 2022 international dollars.

| **Year** | **Total annual revenue** | **Total direct costs** | **Gross profit** | **Gross margin** | **Total indirect costs** | **Net profit** | **Net margin** | **VAT** |
| --- | --- | --- | --- | --- | --- | --- | --- | --- |
| 2018 | 69008 | 77954 | -8946 | -13% | 70558 | -79505 | -115% | 12421 |
| 2019 | 97160 | 136986 | -39826 | -41% | 354255 | -394080 | -406% | 17489 |
| 2020 | 236177 | 249265 | -13088 | -6% | 335154 | -348242 | -147% | 42512 |
| 2021 | 264851 | 302696 | -37844 | -14% | 272799 | -310643 | -117% | 47673 |
| 2022 | 375676 | 343522 | 32154 | 9% | 216825 | -184671 | -49% | 67622 |

**Table S1h. Pit Vidura modelled average direct cost per job organised by emptying method and customer type when operating at a scale (13,188 jobs completed by 19 exhauster trucks: 4 large, 4 medium, 4 small, and 7 dedicated to semi-mechanical emptying) to replace pit sealing and informal manual emptying in Kigali with semi-mechanical using a cross-subsidy from mechanical emptying.** All financial values are 2022 international dollars. Sludge transfer trips are the proportion of trips to the dumpsite completed to transfer sludge emptied by other vehicles.

| **Vehicle** | **Large exhauster truck** | | **Medium exhauster truck** | | **Small exhauster truck** | | **Semi-mechanical** |
| --- | --- | --- | --- | --- | --- | --- | --- |
| **Customer type** | **Corporate** | **Household** | **Corporate** | **Household** | **Corporate** | **Household** | **Household** |
| Fuel | 161 | 126 | 52 | 52 | 20 | 21 | 21 |
| Repair and maintenance | 51 | 40 | 30 | 30 | 8 | 8 | 16 |
| Vehicle depreciation | 18 | 14 | 16 | 16 | 25 | 26 | 53 |
| Vehicle rental | 33 | 26 | 0 | 0 | 2 | 2 | 2 |
| Emptiers' wages | 23 | 18 | 24 | 24 | 6 | 8 | 41 |
| Dumping fees | 18 | 14 | 5 | 5 | 1 | 1 | 1 |
| Drivers' salaries | 9 | 7 | 7 | 7 | 8 | 8 | 17 |
| Consumables | 3 | 2 | 3 | 3 | 2 | 2 | 5 |
| Equipment | 2 | 2 | 2 | 2 | 4 | 4 | 8 |
| Other | 2 | 2 | 8 | 8 | 3 | 4 | 4 |
| Sludge transfer | - | - | - | - | 61 | 61 | 24 |
|  |  |  |  |  |  |  |  |
| Average direct cost per job | 319 | 250 | 145 | 145 | 140 | 145 | 191 |
| Average revenue per job | 506 | 436 | 232 | 183 | 165 | 203 | 51 |
| Average gross profit per job | 187 | 186 | 87 | 38 | 24 | 57 | -139 |
| Average gross margin | 37% | 43% | 38% | 21% | 15% | 28% | -270% |
| Number vehicles | 4 | 4 | 4 | 4 | 4 | 4 | 7 |
| Sludge transfer trips | 26% | 26% | 0% | 0% | 0% | 0% | 0% |
| Total annual jobs | 1497 | 981 | 354 | 3636 | 197 | 3024 | 3500 |
| Total VAT | 136396 | 76951 | 14757 | 120011 | 5835 | 110275 | 32412 |

**Table S1i. Total financial cross-subsidy required per year to replace pit sealing in Kigali.** All financial values are 2022 international dollars

| **Symbol** | **Variable** | **Value** | **Unites** | **Notes** |
| --- | --- | --- | --- | --- |
| a | Average full use direct cost per job for semi-mechanical emptying | 191 | Int$ | - |
| b | Tariff for households to use formal semi-mechanical emptying services | 51 | Int$ | From literature^a^ |
| c | Kigali total population | 1,745,555 | people | From literature^c^ |
| d | Proportion of households using shared pit-latrines with constructed floor slabs | 46.90% | - | From literature^c^ |
| e | Proportion of households using pit-latrines that report emptying or sealing | 87% | - | From literature^a^ |
| f | Average low-income household size | 6.1 | people | From literature^a^ |
| g | Numbers households sharing a pit-latrine | 4 | households | From literature^b^ |
| h | Average household pit-latrine emptying frequency | 8.7 | years | From literature^a^ |
| **FCS** | **Total financial cross-subsidy required per year to replace pit sealing** | **466867** | **Int$ per year** | **FCS=(a-b)(c.d.e)(f.g.h)^-1^** |

^a^Burt Z, Sklar R, Murray A. Costs and Willingness to Pay for Pit Latrine Emptying Services in Kigali, Rwanda. Int J Environ Res Public Health. 2019;16(23):4738.

^b^Tsinda A, Abbott P, Pedley S, Charles K, Adogo J, Okurut K, et al. Challenges to Achieving Sustainable Sanitation in Informal Settlements of Kigali, Rwanda. International Journal of Environmental Research and Public Health. 2013;10(12):6939-54.

^c^NSIR. Fifth Population and Housing Census 2022. Kigali, Rwanda: National Insitute of Statistics of Rwanda; 2022.
