# Supplementary material for: Cross-subsidies are a viable option to fund formal pit latrine emptying services: Evidence from Kigali, Rwanda
Source: PLoS One. 2024 Aug 22;19(8):e0307471. doi: 10.1371/journal.pone.0307471 (PMC11341047; doi:10.1371/journal.pone.0307471)
Supplement: Attachment — Submitted filename: Response to Reviewers.docx [file pone.0307471.s004.docx]

University of Leeds, 24 June 2024

Dear Dr Ahmed Mancy Mosa,

Thank you for identifying reviewers and coordinating their responses to review this manuscript. Please see below my responses to the points raised by yourself and the two reviewers.

I also included the paper in my PhD thesis and the examiners made two specific comments which I have addressed in the manuscript and included at the end of this letter.

Sincerely, Jonathan Wilcox

**Editor comments**

1. *When submitting your revision, we need you to address these additional requirements. Please ensure that your manuscript meets PLOS ONE's style requirements, including those for file naming.*

Confirmed.

2. *Please include a complete copy of PLOS’ questionnaire on inclusivity in global research in your revised manuscript.*

I have completed the questionnaire (please see updated submission) and include a sentence in the ‘Methods’ section detailing that permission was provided by the service provider which collected the data for it to be used in the study.

**Reviewer 1 comments**

3. *Abstract: The abstract reads well for the most part. I particular, I suggest adding a couple of sentences highlighting the contributions of this work to the state of the art and the major potential implications from the conducted research.*

I have added a sentence to the abstract highlighting the specific gap to imply the contribution that is made i.e. that there are no academic studies assessing this funding mechanism.

I have added a sentence highlighting the major implication of the findings (that cross subsidies are a viable funding mechanism).

4. *The overview of the relevant literature seems to be quite short. Please double check for the most recent and relevant studies published over the last 2-3 years. It might be a good idea to allocate a separate section, which is specifically devoted to the literature review.*

In the second paragraph of the introduction I summarise the relevant literature and identify a research gap based on recent academic and grey literature. This includes references from every year since 2016.

PLOS guidance for introductions is to include a “brief review of the key literature” which I believe the second paragraph satisfies.

5. *Please make sure that all the adopted assumptions are supported by the relevant references. This will help justifying the adoption of these assumptions.*

The modelling assumptions are already supported by eight references where values are taken from literature. Are there specific concerns about any of the assumptions made?

6. *Research Method: Please provide a more thorough discussion in order to better justify the adopted of the selected research* *method. More supporting references might be helpful to the future readers.*

I have added an additional paragraph to the beginning of the ‘Modelling assumptions’ section to introduction the overall modelling method.

7. *Future research: Please provide more details regarding the limitations of this study. Then, it would be good to create a more detailed discussion regarding the future research needs. I suggest listing a set of bullet points for the future research needs. This would look more appealing to the future readers.*

The ‘Limitations’ section is ~200 words long. What further details are required? I have added an additional sentence regarding generalising results to other contexts.

The future research needs are included in the conclusion.

**Reviewer 2 comments**

8. *It’s recommended to include some data results in the abstract.*

I added a financial value for the cross-subsidy required. I chose to exclude other numeral results (e.g. profit margins, revenue or costs) because they are context specific.

9. *The context of your study should be placed in the Introduction section. In the Methods and Materials section, only the research methodology, modelling, and data analysis methods are detailed.*

I included details about the study context in the method section as this was the format adopted by a previous paper in PLOS (Peletz et al, 2020, When pits fill up: Supply and demand for safe pit-emptying services in Kisumu, Kenya)

10. *Have you considered the transport path optimization to reduce the cost?*

No. This is a good point. I have added a sentence to the limitations section of the discussion and included two references.

11. *Differences in fuel consumption exist between different models of trucks, even for the same type of truck mentioned in the manuscript, such as large exhauster trucks. Should you discuss this in the manuscript?*

The difference in fuel consumption between vehicles is highlighted in the ‘Methods and materials’ section (lines 107-108), in the ‘Results’ section (line 277), and in the ‘Discussion’ section (line 448).

12. *In Figure 3, it looks like the "Chart Area" should not appear in the figure, please re-upload it.*

This has been corrected.

13. *There are many assumptions in the model. Is it still applicable and compatible in real cases?*

Are there any specific concerns with any of the assumptions?

Whilst there are several assumptions, this is also the only study I am aware which uses empirical data from a service provider, and data that is based on operational records, rather than self-reported estimates from service providers. Perhaps this was not made sufficiently prominent in the introduction?

I have added an additional sentence to abstract stating the value of the data, and the contribution this makes.

**PhD examiner comments**

14. *Clarify what a social enterprise is and whether it is allowed to generate profit. Elaborate then how this would be representative of for-profit companies providing the service.*

I have added a sentence (lines 94-96) explaining the term social enterprise.

15. *Correct the reference to costs not being considered for emptying requests that were not converted to completed jobs*

Lines 144-145 were added to describe how the costs for unconverted jobs are included in analysis.
